# Gender differences in the relationship between informal caregiving and subjective health: the mediating role of health promoting behaviors

**DOI:** 10.1186/s12889-022-12612-3

**Published:** 2022-02-15

**Authors:** Aeri Kim, Kyungmi Woo

**Affiliations:** 1 Center for Human-Caring Nurse Leaders for the Future By Brain Korea 21 (BK 21) four project, College of Nursing, Seoul National University, 103 Daehak-ro, Jongno-gu, Seoul, Republic of Korea 03080; 2 College of Nursing, Research Institute of Nursing Science, Seoul National University, 103 Daehak-ro, Jongno-gu, Republic of Korea Seoul, 03080

**Keywords:** Informal caregiver, Subjective Health, Self-rated Health, Health Satisfaction, Health Promoting Behaviors, Longitudinal study, General estimating equations, Fixed effects, Gender difference, KLoSA

## Abstract

**Background:**

In most of developed societies, the prevalence of informal care is on the rise due to rapid population ageing. This study investigates longitudinal associations between informal caregiving and health among caregivers and potential gender differences in this relationship. Moreover, drawing on the Health Promotion Model, this study examines the mediating role of health promoting behaviors in the link between informal caregiving and caregiver’s health.

**Methods:**

Seven waves of a large-scale (*N* = 9,608), a nationally representative longitudinal study of middle- and old-aged adults in Korea between 2006 and 2018, were used. To address the possibility of omitted variable bias, this study employed ordinary least squares models with lagged dependent variables (OLS-LDV) as well as fixed effects (FE) models. Univariate Sobel-Goodman mediation tests were used.

**Results:**

Findings from OLS-LDV models showed that transition into informal caregiving is negatively associated with health satisfaction and self-rated health. FE results also suggest that our results are robust to controlling for unobserved heterogeneity. In the model where informal caregiving is interacted with gender, we found that these associations were largely driven by women caregivers. Results from Sobel-Goodman tests revealed that a decrease in regular exercise partially explains the observed association between informal caregiving and subjective health outcomes (11% for health satisfaction and 8% for self-rated health).

**Conclusions:**

Although informal caregiving can be a rewarding role, it poses a threat to caregiver’s subjective health. Findings of this hold important implications and provide evidence in support of a gender-conscious approach to improve the health and well-being of informal caregivers.

**Supplementary Information:**

The online version contains supplementary material available at 10.1186/s12889-022-12612-3.

## Background

Informal caregivers are individuals who provide unpaid assistance and care to family members, friends, or other people in need of support doing everyday tasks [1]. In South Korea (hereafter Korea), the demand for caregiving is on the rise as the Korean population is rapidly aging and has a high prevalence of chronic diseases and dementia among older adults [1, 2]. In Korea, 89.4 percent of older adults who require help to meet their self-care needs rely on family members [3].

Although informal caregivers play a crucial role in promoting the health of care recipients, caregiving is also known to have adverse effects on the health of caregivers [4]. A large body of literature has documented that informal caregivers experience a higher prevalence of psychological distress and depression compared with individuals who are not caregivers [5, 6]. Engaging in informal caregiving also heightens the risk of a variety of unfavorable physical health outcomes such as mortality, coronary heart disease, and poor self-reported health [7–10].

The Health Promotion Model, proposed by Pender and colleagues [11], provides important insights into how informal caregiving may harm caregiver health and well-being. While caring for a family member can be rewarding, caregiving involves physically and psychologically demanding tasks that can cause caregiving burdens such as fatigue, stress, and pressure [12]. To ease feelings of stress associated with caregiving, informal caregivers may engage in unhealthy behaviors such as smoking and drinking [13, 14], which in turn lead to poor health.

Moreover, when providing care, informal caregivers are likely deprived of the opportunity and time to take care of their own health [15]. Due to time constraints, informal caregivers are less likely to exercise and spend less time exercising than their non-caregiver counterparts [16]. Caregivers also tend to develop unhealthy dietary habits, such as having irregular meals or eating unhealthy foods [17]. These unhealthy behaviors may put caregivers at the risk of developing obesity [9, 18]. In addition, caregiving may negatively affect the health of informal caregivers through delays in seeking or failure to seek healthcare (e.g., routine health check-ups, adherence to medical care and treatments, health screenings, etc.) [19].

Despite a growing body of evidence on the link between being an informal caregiver and health [8], three knowledge gaps remain. First, it is unclear whether the association between informal caregiving and health is confounded by unobserved heterogeneity at the individual level. Given that previous studies primarily rely on cross-sectional data from smaller regional samples and do not fully control for time-invariant factors, they do not rule out the possibility that individual heterogeneity may spuriously drive observed associations [20, 21]. Taking into account this potential source of bias may explain some conflicting results from prior studies about the health consequences of informal caregiving [22].

Second, few studies have directly tested potential mechanisms, such as health promoting behaviors, connecting informal caregiving to health. Two notable exceptions are works by Gallant and Connell [23] and Acton [24], both of which showed that health promoting behaviors partially mediate the association between informal caregiving and caregiver health. While interesting, however, both studies are limited in that they used data from non-representative, cross-sectional samples and included a limited set of controls in their models. Moreover, although it is crucial to consider gender differences under the Health Promoting Model [25], none of two previous studies the possibility of gender differences in the mediating role of health promoting behaviors in the relationship between caregiving status and subjective health.

Third, despite existing evidence on gender differences in the health consequences of informal caregiving, further evidence is needed. In fact, the findings from a meta-analysis suggest that women caregivers including wives, daughters, and daughters-in-law tend to report worse health than caregivers who are men [26]. Such gender differences were found consistently in both high-income [27, 28] and low-/middle-income countries [29]. However, some studies especially on dementia caregivers found no gender differences in caregivers’ health [30, 31]. Moreover, given that a large body of previous studies on caregivers’ health relied on cross-sectional datasets, it may be important to confirm gender differences in caregivers’ health in more rigorous longitudinal models with a nationally representative sample.

Using seven waves (that span 12 years) of a large-scale, nationally representative longitudinal study of middle- and old-aged adults in Korea, this study fills the abovementioned gaps in extant research. To examine whether informal caregiving is associated with caregivers’ subjective health, measured by health satisfaction (HS) and self-rated health (SRH), this study employs two longitudinal models that are based on different assumptions and have different advantages and disadvantages [32, 33]. This study first estimates OLS regression models with lagged dependent variables (OLS-LDV). To gauge the extent of potential bias related to unobserved heterogeneity at the individual level, this study also estimates fixed effects (FE) models.

**Fig. 1:**
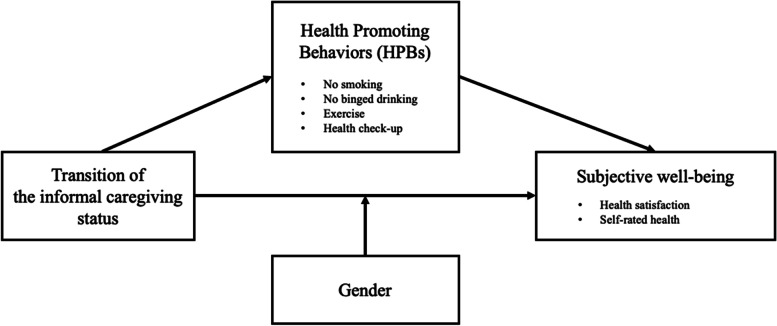
A modified research framework from Pender's Health Promoting Model (1996)

Using longitudinal models, this study examines whether there are gender differences in the relationship between informal caregiving and caregivers’ subjective health. More specifically, an interaction term of informal caregiving status and a female indicator is added in the regression model to assess potential gender differences. We also provide results from gender-stratified models. This study also investigates whether health promoting behaviors mediate the association between informal caregiving and caregivers’ subjective health. Univariate Sobel-Goodman mediation tests are used to gauge the extent to which each health promoting behavior explains the observed association between informal caregiving and health. This study considers the following health promoting behaviors as potential mediators: smoking, binge drinking, physical exercise, and health check-up status [34] (Fig. 1).

## Data and methods

### Data

This study uses a nationally representative longitudinal data set from Korean Longitudinal Study of Aging (KLoSA), collected and provided by Korea Employment Information Service [35]. The survey began in 2006 and followed a multi-stage stratification sampling (region and housing type) based on enumeration districts from the Population and Housing Census of Korea Statistics. Trained interviewers visited selected households and computer assisted personal interviews was conducted with adults aged over 45 (born in 1961 or earlier) every two years. The KLoSA was created to capture socioeconomic, psychological and physical health, and demographic information that could inform policies relevant to the middle- and older-aged individuals in Korea.

The present study uses data from Wave 1 (2006) to Wave 7 (2018). Of 10,254 respondents surveyed in Wave 1, the retention rate was 86.6% (8,875) at Wave 2, and 78.8% (7,491) of participants remained in the study through Wave 7. Dropping those with missing values on key variables yields a final analytic sample of 9,608 individuals (5,419 women and 4,189 men), and results in 42,507 person-years. Each respondent contributes an average of about 4.42 person-years to our panel data. This study was reviewed and approved by the Institutional Review Board at Seoul National University (No. E2102/003–007). This study was conducted in accordance with the Declaration of Helsinki ethical principles for research involving human data. Trained interviewers obtained informed consent from enrolled participants.

### Measures

#### Dependent variables

This study uses two dependent variables to capture an individual’s subjective health status: health satisfaction (HS) and self-rated health (SRH). The measure of HS was assessed with the following question: “How satisfied are you with your health compared with other individuals who are your same age?” HS was measured on a scale ranging from 0 to 100. SRH is a measure of individuals’ perceptions about multidimensional aspects of their health status including psychological, physical, and social health [36]. SRH was measured by the following question: “How would you rate your current health in general?” The five response categories include: “very good”, “good”, “fair”, “poor”, and “very poor”. We dichotomized the responses into “poor (fair, poor, and very poor) health” (0) versus “good (good and very good) health” (1).

#### Independent variable

Informal caregiving status was measured by the following questions: “Did you provide help or assistance in activities of daily living (ADL) such as eating, dressing, bathing, toileting, grooming and getting around during the last 12 months to your family members and/or other relatives aged over 10 regardless of co-habitation?” and “How many hours did you spent per week to provide help or assistance for the care-recipient during the last 12 months?”. Since there is no formal threshold related to the amount of hours of informal care needed to classify individuals as informal caregivers, we considered individuals informal caregivers if they spend at least 7 h per week providing ADL support and assistance [37]. The mean hours of informal care per week was 30.86 h (standard deviation = 30.99) among those who provided ADL assistance at least one hour per week.

This study relies on OLS models with a lagged dependent variable. This approach takes advantage of the longitudinal nature of the data. This study captured change in and continuity of the status of providing informal care between two waves. The measure of informal caregiving comprises of the following four mutually exclusive dummy variables: (1) no caregiving at any waves (No to No, never caregivers); (2) no caregiving at Wave t, caregiving at Wave t + 1 (No to Yes, started CG); (3) caregiving at Wave t, no caregiving at Wave t + 1 (Yes to No, stopped CG); and (4) caregiving at both waves (Yes to Yes, continued CG). The first and second groups were compared to analyze whether starting to provide care (between two waves) leads to change in one’s subjective health.

#### Mediating variables

This study considers the following potential mediators: smoking, binge drinking, physical exercise, and health check-ups [34]. All these variables are time-varying. We capture smoking using a dichotomous variable indicating whether the person self-reported that they are a current smoker (0) or not (1). Binge drinking was based on respondents’ agreement with five statements about drinking habits. We coded this variable as 1 if respondents did not agree with any of the statements and 0 if they agreed with at least one statement. Exercise was measured by the number of days the respondent engages in physical activity in a given week. The measure of health check-ups was coded 1 if the respondent had a health check-up in the past two years; 0 otherwise. Note that all proposed mediating variables were coded such that higher scores represent a favorable outcome.

#### Control variables

This study includes a large set of control variables. Time-constant control variables include gender, age, education, and number of children. The education variable was grouped into the following four categories: below elementary, middle school, high school, and college or higher. Time-varying control variables include marital status, household size, household income, home ownership, economic activity, region of residence, and number of diagnosed chronic diseases. Marital status was measured by whether the respondent was married or not (single, widowed, divorced, or separated). Household income was grouped into quartiles for each wave. Home ownership was assessed by whether the respondent owned a house or not. Economic activity indicates whether the respondent was economically active. In this study, “Economically active” person included those who were working for payment such as an employee or a self-employee or working for family business over 18 h per week at the survey point. Place of residence was also controlled for and is coded as a categorical variable with the following categories: large city, small city, or rural area. The number of chronic diseases an individual has was the sum of doctor-diagnosed chronic diseases including hypertension, diabetes mellitus, cancer or a malignant tumor, chronic lung disease, cerebrovascular disease, arthritis or rheumatoid arthritis, psychological disease, liver disease, and prostatic disease.

### Statistical Analysis

This study adds rigor to extant research on informal caregiving, health promoting behaviors, and subjective health. More specifically, we employ OLS models with lagged dependent variables (OLS-LDV) and fixed effects (FE) models. Each of these approaches provides useful and distinct information. To account for the fact that our data have repeated measures, generalized estimating equations (GEE) are used in OLS-LDV models [38]. The OLS-LDV model predicts subjective health in Wave t + 1, while controlling for subjective health at Wave t (lagged dependent variable) as well as a wide range of control variables measured at Wave 1. The estimation model can be expressed as follows:

1 $$g\left(E\left[{Y}_{i,t+1}\right]\right)=\alpha +\lambda {Y}_{i,t}+{\beta }_{1}{CG01}_{i}+{\beta }_{2}{CG10}_{i}+{\beta }_{3}{CG11}_{i}+{{\varvec{X}}}_{i}\gamma +{{\varvec{Z}}}_{i,t}\delta +{\theta }_{t}$$

In Eq. (1), ﻿g is a link function determined by the form of the dependent variable. In our analyses, ﻿the identity link function is specified. We assume an exchangeable correlation structure for the clusters with robust standard errors [39]. In supplementary analyses, we confirmed that results from the regression model with autoregressive correlation are substantially similar to the results reported in this paper (Table S1-S3 in online supplementary material).

${\mathrm{Y}}_{\mathrm{i},\mathrm{t}+1}$ is subjective health (HS or SRH) measured at Wave t + 1. $\mathrm{CG}01$, $\mathrm{CG}10$, and $\mathrm{CG}11$ are the categorical variables which express groups sorted by transition of informal caregiving status (CG00 is reference group, never caregivers). The first and second digits of the number following $\mathrm{CG}$, indicate whether an individual was a caregiver in Wave t and Wave t + 1, respectively. For example*, in *${CG10}_{i}$*,* the subscript refers to individuals who had provided informal care at wave t and stopped at wave t + 1. To assess the association between informal caregiving and caregiver’s subjective health, we focus particularly on ${\beta }_{1}$, the coefficient of ${CGxx}_{i}$, because it captures whether individuals who started to provide caregiving between the two waves experience a decrease in subjective health compared with their never caregiver counterparts. To examine gender differences in the relationship between informal caregiving status and subjective health, we introduce interaction terms of caregiving status and gender.

To remove bias associated with selection into caregiving and to better address the issue of temporal ordering between informal caregiving and subjective health, we include a lagged value of the dependent variable (${Y}_{i,t}$) by one survey period in this OLS-LDV model. Individual-level characteristics were added in order to reduce the possibility that this source of bias drives our results. The vector ${{\varvec{X}}}_{i}$ represents a set of time-constant covariates, and the vector ${{\varvec{Z}}}_{it}$ represents a set of time-varying covariates. In all models, survey years (${\theta }_{t}$) are adjusted for.

To further gauge the extent to which unobserved individual heterogeneity confounds the association between informal caregiving and caregivers’ subjective health, this study estimates FE models. FE models are useful in eliminating bias due to time-invariant unobserved heterogeneity because they take into account observed and unobserved time-constant variables within-groups in panel data [32, 33]. FE models are specified as follows:

2 $${Y}_{it}=\alpha +\beta {CG}_{it}+{{\varvec{Z}}}_{it}\tau +{\theta }_{t}+{u}_{i}+{\varepsilon }_{it}$$

$\mathrm{In Eq}. \left(2\right), {u}_{i}$ represents individual fixed effects. In order to reduce potential bias arising from time-varying unobserved individual heterogeneity, the model controls for time-varying confounding factors (${{\varvec{Z}}}_{it}$) and survey years. FE models control for preexisting, stable individual differences, for example, personality traits that may jointly predict becoming an informal caregiver, HPBs, and subjective health [32, 33]. Although FE models are useful to remove unobserved heterogeneity, an important weakness is that they are not ideal to handle a potential reverse causation issue. In contrast, OLS-LDV models better address the temporal ordering of the dependent and independent variables [40], by explicitly comparing later subjective health status among individuals who remained in non-caregiving status and transitioned into informal caregiving status. Therefore, while we use FE models as a supplementary model to examine whether unobserved heterogeneity is a serious problem, we rely on OLS-LDV models for mediation analyses.

To explore the mediating role of health promoting behaviors (HPBs) between transition into informal caregiving and subjective health, we conduct Sobel-Goodman mediation tests [41]. To implement Sobel-Goodman test, we estimate the relationship between the independent variable (changes in informal caregiving status between Wave t and Wave t + 1) and the mediating variable (health promoting behaviors at Wave t + 1), as well as the relationship between the mediating variable and the dependent variable (subjective health at Wave t + 1).

Lastly, it is worth noting that, to ease interpretation, we use linear probability models for the binary measure of self-rated health [42]. Moreover, linear probability models are preferred in this study because exceedingly complicated statistical models (e.g., path-analytic decomposition for mediation and estimation of cross-partial derivative for moderation) should be fit to formally test moderation and mediation in nonlinear models such as logistic regression models [43–46]. However, supplementary analyses confirmed that results from logistic regression models were qualitatively similar to our original results (Table S4 in online supplementary material). For all statistical analyses, we used STATA, version SE 16.1(Stata Corp., College Station, TX).

## Results

Table 1 presents summary statistics of 8,520 participants were those who had no missing values in key variables in both Wave 1 and Wave 2. The sample for summary statistics differs from the final sample size of 9,608 because our data analysis relies on unbalanced panel data. Statistically significant gender differences (*p* < 0.05) were found for a number of variables, including HS and SRH. The average score for HS was 52.7 for women and 58.3 for men. About 28.8% of women reported having good health, whereas 42.8% of men reported having good or very good health. The majority of respondents were not caregivers in either wave. Less than 1% of the analytic sample were continued CG. About 3% of respondents changed caregiving status between the two waves. Although given the seemingly small percent of informal caregivers at wave 1, the use of 7 waves of longitudinal data reduces our concerns regarding low statistical power. For example, 865 person-years of observations transitioned between different caregiving statuses across 7 waves (360 for transition into CG, 505 for transition out of CG, and 137 for continued CG). About 56.5% of respondents were women. Average respondent age was about 61.43 years old in 2006.

**Table 1:** Summary statistics, KLoSA (*N* = 8,520)

	Full				Women	Men	Gender
	Mean or Prop	*(SD)*	Min	Max	Mean or Prop	Mean or Prop	diff
***Dependent variables (Subjective health)***							
Health satisfaction (2008)	55.163	*(21.952)*	0.0	100.0	52.740	58.309	*
Self-rated health (2008)	0.349		0.0	1.0	0.288	0.428	*
***Independent variable***							
Informal caregiving (2006–2008)							*
Never caregiver (No to No)	0.966		0.0	1.0	0.962	0.972	
Started CG (No to Yes)	0.010		0.0	1.0	0.011	0.010	
Stopped CG (Yes to No)	0.018		0.0	1.0	0.021	0.015	
Continued CG (Yes to Yes)	0.006		0.0	1.0	0.007	0.004	
***Mediating variables***							
No smoking	0.810		0.0	1.0	0.967	0.605	*
No binge drinking	0.908		0.0	1.0	0.977	0.819	*
Exercise	1.735	*(2.535)*	0.0	7.0	1.594	1.918	*
Health check-up	0.539		0.0	1.0	0.531	0.550	
**Control variables**							
Lagged self-rated health (2006)	0.379		0.0	1.0	0.313	0.465	*
Lagged health satisfaction (2006)	56.358	*(24.519)*	0.0	100.0	53.100	60.588	*
Woman	0.565		0.0	1.0	1.000	0.000	
Age	61.437	*(10.808)*	45.0	97.0	61.727	61.061	*
Educational level							*
Elementary or lower	0.468		0.0	1.0	0.585	0.317	
Middle school	0.163		0.0	1.0	0.159	0.169	
High school	0.270		0.0	1.0	0.213	0.344	
College or higher	0.099		0.0	1.0	0.044	0.170	
Number of children	3.019	*(1.508)*	0.0	10.0	3.127	2.880	*
Marital status							*
Single, widowed, divorced, or separated	0.208		0.0	1.0	0.309	0.077	
Married	0.792		0.0	1.0	0.691	0.923	
Household size	2.973	*(1.354)*	1.0	11.0	2.900	3.067	*
Household income quartile							*
Q1	0.231		0.0	1.0	0.246	0.212	
Q2	0.254		0.0	1.0	0.257	0.250	
Q3	0.272		0.0	1.0	0.258	0.290	
Q4	0.171		0.0	1.0	0.160	0.186	
Missing	0.072		0.0	1.0	0.080	0.062	
Home owner	0.781		0.0	1.0	0.770	0.794	*
Economic activity	0.395		0.0	1.0	0.248	0.587	*
Place of residence							
Large city	0.434		0.0	1.0	0.441	0.425	
Small city	0.321		0.0	1.0	0.315	0.329	
Rural	0.245		0.0	1.0	0.244	0.245	
Number of chronic diseases	0.747	*(0.953)*	0.0	7.0	0.805	0.670	*
Observations	8,520				4,813	3707	

*Note.* Summary statistics are based on 2006 data unless otherwise indicated. * Differences between women and men are statistically significant, *p* < 0.05. Chi-squared tests for categorical variables and *t* tests for continuous variables were performed. *CG* Caregiver

Table 2 presents coefficients from OLS-LDV and FE models that regressed subjective health on caregiving transitions, as well as an array of covariates. Models 1 and 2 present results for the pooled sample. Regression models are also estimated separately for women (Models 3 and 4) and men (Models 5 and 6). The odd-numbered models show results from OLS-LDV regression models that control for a set of observed time-constant and time-varying covariates, as well as a lagged dependent variable. The even-numbered models present results from FE models. Panel A presents results for HS and Panel B for SRH.

**Table 2:** Regression of subjective health on informal caregiving, By gender and by estimation model

Gender	Full		Women		Men	
Estimation model	GEE	FE	GEE	FE	GEE	FE
	DV	DV	DV	DV	DV	DV
	at t + 1	at t	at t + 1	at t	at t + 1	at t
	Model 1	Model 2	Model 3	Model 4	Model 5	Model 6
***Panel A. DV* = *Health satisfaction***						
* Change in informal caregiving between t and t* + *1 (Ref: No to No)*						
Started CG (No to Yes)	-1.857^*^		-3.236^**^		0.603	
	[-3.582,-0.132]		[-5.318,-1.154]		[-2.383,3.588]	
Stopped CG (Yes to No)	-1.174		-0.590		-2.148	
	[-2.735,0.388]		[-2.431,1.252]		[-4.993,0.696]	
Continued CG (Yes to Yes)	-0.865		-1.948		0.815	
	[-4.201,2.471]		[-6.713,2.817]		[-3.457,5.087]	
Informal caregiving status at t						
Yes		-1.997^*^		-3.800^***^		1.707
		[-3.707,-0.287]		[-5.927,-1.672]		[-1.054,4.467]
N	9,606	9,606	5,418	5,418	4,188	4,188
Observations	42,482	42,482	24,135	24,135	18,347	18,347
***Panel B. DV* = *Self-rated health***						
* Change in informal caregiving between t and t* + *1 (Ref: No to No)*						
Started CG (No to Yes)	-0.049^*^		-0.063^**^		-0.022	
	[-0.086,-0.011]		[-0.109,-0.018]		[-0.087,0.044]	
Stopped CG (Yes to No)	0.001		0.003		0.002	
	[-0.033,0.034]		[-0.037,0.042]		[-0.057,0.061]	
Continued CG (Yes to Yes)	-0.080^**^		-0.120^***^		-0.018	
	[-0.136,-0.025]		[-0.180,-0.060]		[-0.116,0.081]	
Informal caregiving status at t						
Yes		-0.031		-0.059^**^		0.019
		[-0.066,0.004]		[-0.099,-0.018]		[-0.045,0.083]
N	9,608	9,608	5,419	5,419	4,189	4,189
Observations	42,507	42,507	24,148	24,148	18,359	18,359
Time-constant control variables	Yes	No	Yes	No	Yes	No
Time-varying control variables	Yes	Yes	Yes	Yes	Yes	Yes
Lagged dependent variable	Yes	No	Yes	No	Yes	No

^*^ *p* < 0.05; ** *p* < 0.01; *** *p* < 0.001

*Note*. Robust standard errors in GEE models and clustered standard errors in FE models were used. In the models, survey years were adjusted for. Time-constant control variables include gender, age, education, and number of children. Time-varying control variables include marital status, household size, household income, home owner, economic activity, region of residence, and number of chronic diseases. *GEE* Generalized Estimating Equation, *FE* Fixed Effects, *DV* Dependent Variable, *CG* Caregiver

In Table 2, Model 1 shows that transitioning into or becoming an informal caregiver is associated with a decrease in subjective health, even after adjusting for a large set of covariates as well as a lagged dependent variable (*b* = -1.857 for HS and *b* = -0.049 for SRH). However, the subjective health of individuals who transitioned out of being an informal caregiver was not different from the subjective health of individuals who were never caregivers during the study period. Continuing to be an informal caregiver is negatively associated with SRH (*b* = -0.080, Panel B), but not HS. In Model 2, the FE estimates suggest that there is a negative association between providing care and HS and that this association is robust to controlling for unobserved time-invariant characteristics (*b* = -1.997, Panel A). Although the effect of informal caregiving was in the expected direction, the observed negative association for SRH was not statistically significant in FE models (Panel B).

We turn to examination of gender differences in the association between informal caregiving and subjective health. As shown in Models 3 and 4, findings among women were consistent with those for the pooled sample (Models 1 and 2). In fact, the association is even more pronounced. For women, becoming a caregiver between the two waves was negatively associated with subjective health (*b* = -3.236 for HS and *b* = -0.063 for SRH). Similar to the pooled sample, women who provide care continuously showed a decrease in SRH (*b* = -0.120, Panel B), but not HS. The FE estimates are substantially similar to results from OLS-LDV models, suggesting that the negative association between informal caregiving and subjective health is robust among women. In contrast, as shown in Models 5 and 6, OLS-LDV and FE models alike suggest that caregiving is not associated with men’s subjective health.

To examine whether gender differences in the relationship between transition into informal caregiving and subjective health are statistically significant, we run interaction model in which we add interaction terms of caregiving transition status and a female indicator. In Model 1 and Model 2 of Table 3, negative and statistically significant interaction terms (*b* = -4.046 for GEE and *b* = -5.981 for FE models) indicate that gender differences for the association between informal caregiving and health satisfaction found in Table 2 are statistically significant. For self-rated health (Model 3 and Model 4), similar patterns were found, though the interaction term in GEE models is statistically insignificant.

**Table 3:** Regression of subjective health on informal caregiving, by estimation model

Gender	Full			
Estimation model	GEE	FE	GEE	FE
	Health satisfaction	Health satisfaction	Self-rated health	Self-rated health
	at t + 1	at t	at t + 1	at t
	Model 1	Model 2	Model 3	Model 4
*Change in informal caregiving between t and t* + *1 (Ref: No to No)*				
Started CG (No to Yes)	0.721		-0.019	
	[-2.267,3.709]		[-0.085,0.047]	
Stopped CG (Yes to No)	-2.081		0.000	
	[-4.936,0.773]		[-0.059,0.059]	
Continued CG (Yes to Yes)	0.807		-0.018	
	[-3.665,5.279]		[-0.119,0.082]	
Started CG × Female ^a^	-4.046 (0.029)^*^		-0.046 (0.260)	
	[-7.688,-0.403]		[-0.126,0.034]	
Stopped CG × Female ^a^	1.382 (0.425)		0.000 (0.990)	
	[-2.014,4.779]		[-0.071,0.072]	
Continued CG × Female ^a^	-2.844 (0.392)		-0.107 (0.073)	
	[-9.353,3.665]		[-0.224,0.010]	
Informal caregiving status at t				
Yes		1.928		0.019
		[-0.850,4.706]		[-0.045,0.083]
Yes × Female ^a^		-5.981 (0.001)^***^		-0.079 (0.040)^*^
		[-9.486,-2.476]		[-0.155,-0.004]
N	9,606	9,606	9,608	9,608
Observations	42,482	42,482	42,507	42,507
Time-constant control variables	Yes	No	Yes	No
Time-varying control variables	Yes	Yes	Yes	Yes
Lagged dependent variable	Yes	No	Yes	No

^*^ *p* < 0.05; ** *p* < 0.01; *** *p* < 0.001

*Note*. ^a^ reported with p-value in a parentheses. Robust standard errors in GEE models and clustered standard errors in FE models were used. In the models, survey years were adjusted for. Time-constant control variables include gender, age, education, and number of children. Time-varying control variables include marital status, household size, household income, home owner, economic activity, region of residence, and number of chronic diseases. *GEE* Generalized Estimating Equation, *FE* Fixed Effects, *DV* Dependent Variable, *CG* Caregiver

To interpret the estimated coefficient of the association for women (Model 3 of Table 2), compared to never caregivers in both waves, individuals who transitioned into of being an informal caregiver between two waves have on average HS scores that are 3.236 points lower. This difference is about 16% of one-standard-deviation of HS (-3.236/20.575 [overall standard deviation for women in the sample]). In terms of SRH, the probability of having good or very good health for those who became an informal caregiver is 6.3 percentage points lower than that found among never caregivers. Given that 28.8% of women in this sample reported having good or very good health (Table 1), we may deem such difference (about 26% decrease) as substantively significant.

To further assess the effect size for our findings, we use the magnitude of the associations between homeownership and number of chronic diseases as a benchmark to assess the observed effects. The effects of transition into an informal caregiver on health satisfaction are equivalent to about 84% (-3.236/3.856) of the effect of being a homeowner and the effect of having 1.43 chronic diseases (-3.236/-2.266). The effects of transition into an informal caregiver on self-reported health are equivalent to about 233% (-0.063/0.027) of the effect of being a homeowner and the effect of having 1.40 chronic diseases (-0.063/-0.045). Given that one’s homeownership status and health condition are strong predictors of health and well-being, the association between informal caregiving and subjective health seems notable.

In Table 4, to shed light on potential mechanisms that link informal caregiving to subjective health among women, we first estimate the relationship between informal caregiving and a set of mediating variables (smoking, binge drinking, physical exercise, and health check-ups). We present OLS-LDV results separately for the sample of HS (Panel A) and the sample of SRH (Panel B). As expected, the results in Panel A and Panel B are substantially similar due to a significant overlap in the sample. The result shows that transition into informal caregiving is associated with exercise only (Model 3) (*b* = -0.561 for both Panel A and Panel B).

**Table 4:** Regression of mediating variables on informal caregiving, Women only sample

Estimation model: GEE	No smoking at t + 1	No binge drinking at t + 1	Exercise at t + 1	Health check-up at t + 1
	Model 1	Model 2	Model 3	Model 4
***Panel A. Health satisfaction*** *Change in informal caregiving between t and t* + *1 (Ref: No to No)*				
Started CG (No to Yes)	0.003	-0.003	-0.561^***^	-0.020
	[-0.010,0.016]	[-0.019,0.013]	[-0.784,-0.339]	[-0.074,0.034]
Stopped CG (Yes to No)	0.006	0.007^*^	-0.117	-0.029
	[-0.006,0.017]	[0.000,0.013]	[-0.345,0.110]	[-0.075,0.016]
Continued CG (Yes to Yes)	-0.008	-0.008	-0.740^***^	-0.046
	[-0.034,0.017]	[-0.036,0.020]	[-1.166,-0.314]	[-0.156,0.063]
N	5418	5418	5418	5418
Observations	24,135	24,135	24,135	24,135
***Panel B. Self-reported health*** *Change in informal caregiving between t and t* + *1 (Ref: No to No)*				
Started CG (No to Yes)	0.003	-0.003	-0.561^***^	-0.019
	[-0.010,0.016]	[-0.019,0.013]	[-0.783,-0.338]	[-0.074,0.035]
Stopped CG (Yes to No)	0.006	0.007^*^	-0.116	-0.029
	[-0.006,0.017]	[0.000,0.013]	[-0.344,0.111]	[-0.074,0.017]
Continued CG (Yes to Yes)	-0.008	-0.008	-0.740^***^	-0.046
	[-0.034,0.017]	[-0.036,0.020]	[-1.165,-0.314]	[-0.155,0.064]
N	5419	5419	5419	5419
Observations	24,148	24,148	24,148	24,148

^*^ *p* < 0.05; ** *p* < 0.01; *** *p* < 0.001

*Note*. Robust standard errors were used. In the models, survey years were adjusted for. Time-constant control variables include gender, age, education, and number of children. Time-varying control variables include marital status, household size, household income, home owner, economic activity, region of residence, and number of chronic diseases. *GEE* Generalized Estimating Equation, *CG* Caregiver

In Table 5, we estimate the relationship between health promoting behaviors and subjective health whether HPBs mediate the relationship between informal caregiving and subjective health (HS for Panel A and SRH for Panel B) among women. The results suggest that no smoking status and doing exercise are positively associated with both health satisfaction (Panel A) and self-reported health (Panel B). On the other hand, health check-up is associated with an increase in health satisfaction (Panel A), but not self-reported health (Panel B).

**Table 5:** Regression of Subjective health on mediating variables, Women only sample

**Estimation model: GEE**	**Health satisfaction at t + 1**	**Health satisfaction at t + 1**	**Health satisfaction at t + 1**	**Health satisfaction at t + 1**
	**Model 1**	**Model 2**	**Model 3**	**Model 4**
***Panel A. Health satisfaction***				
No smoking at t + 1	2.957^**^			
	[0.894,5.021]			
No binge drinking at t + 1		1.821		
		[-0.512,4.155]		
Exercise at t + 1			0.619^***^	
			[0.510,0.727]	
Health check-up at t + 1				2.852^***^
				[2.227,3.477]
N	5418	5418	5418	5418
Observations	24,135	24,135	24,135	24,135
**Estimation model: GEE**	**Self-reported Health at t + 1**	**Self-reported Health at t + 1**	**Self-reported Health at t + 1**	**Self-reported Health at t + 1**
	**Model 5**	**Model 6**	**Model** **7**	**Model 8**
***Panel B. Self-reported health***				
No smoking at t + 1	0.050^*^			
	[0.012,0.088]			
No binge drinking at t + 1		0.027		
		[-0.024,0.079]		
Exercise at t + 1			0.009^***^	
			[0.007,0.012]	
Health check-up at t + 1				-0.008
				[-0.020,0.004]
N	5419	5419	5419	5419
Observations	24,148	24,148	24,148	24,148

^*^ *p* < 0.05; ** *p* < 0.01; *** *p* < 0.001

*Note*. Robust standard errors were used. In GEE models, survey years were adjusted for. Time-constant control variables include gender, age, education, and number of children. Time-varying control variables include marital status, household size, household income, home owner, economic activity, region of residence, and number of chronic diseases. The table including level of mediating variables in Wave t is available in supplementary materials. *GEE* Generalized Estimating Equation, *CG* Caregiver

**Fig. 2:**
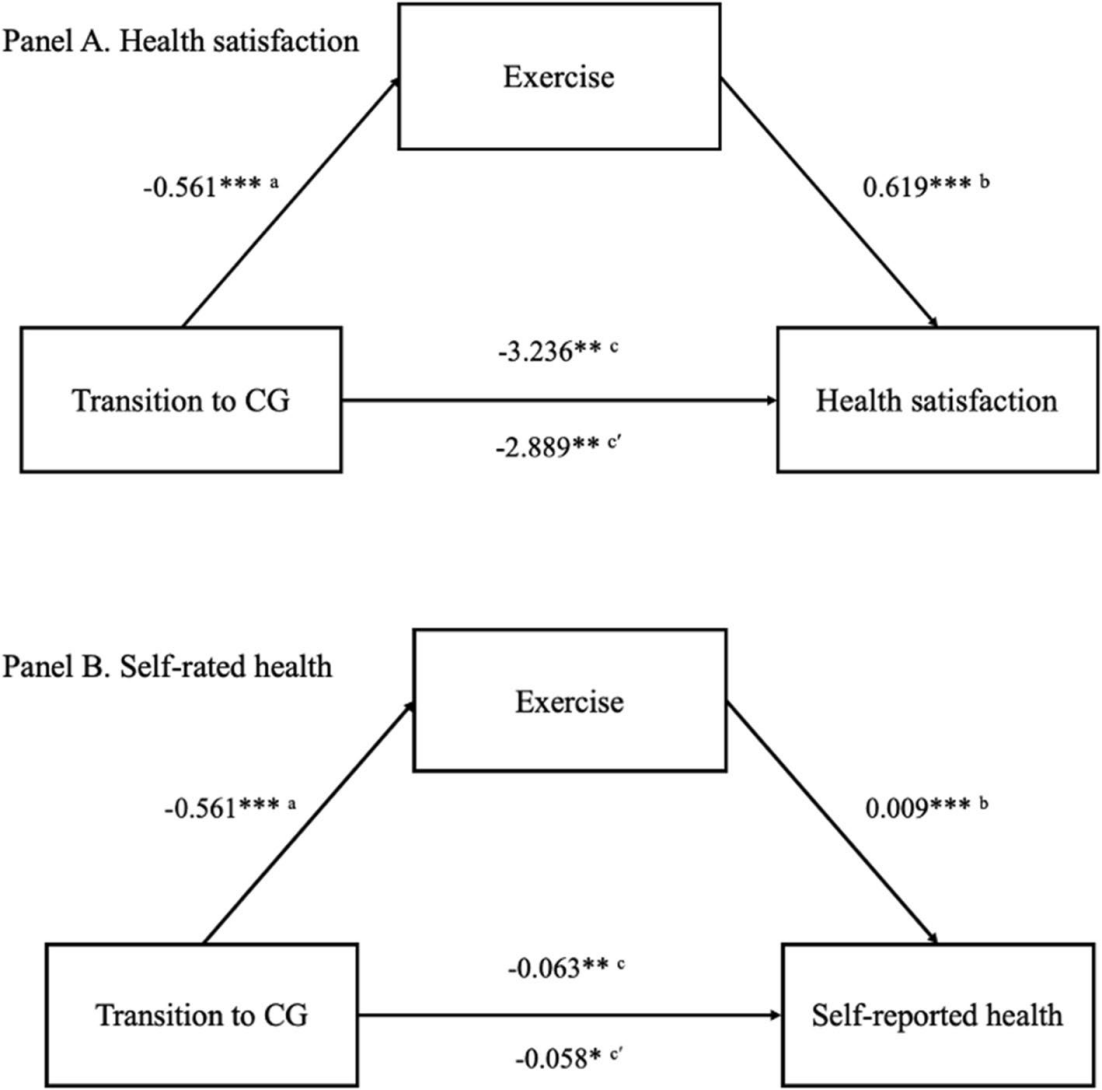
Sobel-Goodman mediation tests. *Note.* Path models for subjective health represents the total effect (c) without the mediator, the direct effect (c′; the values below arrow) via the mediator, and the indirect effect (ab) of the transition into informal caregiving on the subjective health. * *p* < .05, ** *p* < .01. *** *p* < .001

Since exercise is the only variable that is associated with both independent variable (*b* = -0.561, Model 3 of Table 4) and dependent variable (*b* = 0.619 for health satisfaction and *b* = 0.009 for self-reported health, Model 3 of Table 5), we consider it as a potential mediator in Sobel-Goodman mediation tests. In Fig. 2, the results show that the indirect effect of exercise is -0.347 (-0.561*0.619), which is statistically significant at the 1% level. This suggests that adding exercise as a mediator explained about 11% of the association between transition to CG and health satisfaction (from -3.236 to -2.889). Similarly, adding exercise as a mediator explained about 8% of the association between transition to CG and self-reported health (from -0.063 to -0.058). The indirect effect of exercise in this model is -0.005 and is statistically significant at the 1% level. In supplementary analyses, we present results from the models where we sequentially add proposed mediating variables to examine whether the inclusion reduces the estimated coefficient of transition into informal caregiving (Table S5 in online supplementary material).

## Discussion

This study examined the relationship between informal caregiving and subjective health. Using data from a nationally representative longitudinal study of aged 45 and over, we employed two longitudinal analytic models: OLS models with a lagged dependent variable (OLS-LDV) and FE models. The OLS-LDV results of this study showed that individuals who become informal caregivers tend to have lower levels of subjective health compared with those who do not become caregivers in the observed time period. Not surprisingly, this study also found that individuals who continuously engaged in caregiving tend to have even lower levels of SRH than those who started caregiving. This finding is consistent with evidence from previous studies that relied on cross-sectional data [8, 47]. The FE estimates suggest that the negative association between informal caregiving and subjective health is not confounded by unobserved, time-invariant characteristics.

Our results also suggested that the association between informal caregiving and subjective health was largely driven by women. Findings from both OLS-LDV and FE models revealed that informal caregiving is negatively associated with subjective health and that this is the case only among women. No such association was found among men. A joint model with the interaction terms revealed that this gender differences in the relationship between informal caregiving status and subjective health are statistically significant. This finding is consistent with a previous meta-analysis by Pinquart and Sorensen [26]. To put the magnitude of these results in context, we interpret the association as economically significant because the difference in subjective health between women who became a caregiver and remained as a never caregiver is about 16% of one-standard-deviation of HS. The difference in the likelihood of reporting good/very good health between these two groups is 6.3 percentage points.

To explore potential mechanisms undergirding this association, we examined the role of several mediators. Our decision to test their mediating effects was based on Pender’s health promotion model [11], which suggests that being an informal caregiving, as an immediate competing demand, should be considered as the determinants of HPBs. We found that engaging in informal caregiving is only associated with a reduction in exercise among women, but not men. Informal caregiving is not associated with other HPBs and this is true for both women as well as men. Results from mediation analyses showed that a reduction in exercise among women who began to provide care during the study period explains about 11% and 8% of the association between informal caregiving and HS and SRH, respectively. These findings are consistent with Gallent and Connell [23]’s research showing that, among various health promoting behaviors, only physical activity played a major mediating role in the link between neuroticism and depressive symptoms among spousal caregivers. Our results extend the existing literature on gender differences in informal caregivers’ physical health [26] by empirically showing that women caregivers may be worse off than their men counterparts due in part to a reduction in physical exercise. It is, however, worth noting that the magnitude of the remaining association is non-negligible and statistically significant.

Since the KLoSA dataset is from Korea, it might be worthy to know the current state of health promoting behaviors in Korea. According to the reports from OECD in 2019, smoking rates and drinking amounts were in tend to lower than the average of OECD countries [48]. In the case of diet, Korea was one of countries with the highest rate of vegetable consumption [48]. In contrast, while only 19.8% of Korean adults participated in moderate physical activity, the average across 23 OECD countries was 66% [48, 49]. In a study, as in our findings, informal caregivers of cancer patients were revealed less participate in physical activities [50]. In addition, informal caregivers of cancer patients were more like to follow the recommendation of alcohol consumption and conducted cancer screening [50]. However, despite the importance of health promoting behaviors, few studies shed light on the health promoting behaviors of informal caregivers in Korea. Future studies should investigate the health promoting behaviors of informal caregivers to build sufficient empirical evidence.

Our findings about gender differences may be explained by socialized gender roles and gender differences in stress-coping strategies. Previous research about socialized gender roles indicates that women have higher exposure to interpersonal stress events such as providing informal care than men [51]. In fact, among caregivers, women are more likely to take a primary caregiving role and have intense caregiving burdens than men [52, 53]. In addition, gender differences in the subjective health consequences of caregiving may be driven by gendered stress-coping strategies. Women tend to use emotional and avoidance coping strategies to a greater extent than men. Men tend to use relatively more rational coping strategies than women [54]. Thus, among men caregivers, seeking to engage in HPBs may be important even under physical constraints caused by caregiving activities. In contrast, such constraints may be stressors for women caregivers and may harm their health and well-being.

This study has several limitations. First, although our estimation models attempted to account for potential methodological challenges such as reverse causation and confounding, they reduce, but do not entirely remove, these concerns. For example, in OLS-LDV models, we examined whether transition to informal caregiving between time t and t + 1 is related to health condition in time t + 1 while controlling for the base level of health condition in time t. However, this does not fully resolve the issue of reverse causation. In addition, while we controlled for an array of potential confounders in OLS-LDV models and confirm that unobserved time-constant heterogeneity does not seriously affect our results (as shown in FE models), there still remains the possibility of observed and unobserved time-varying confounders at the individual level (e.g., personality, loss of a loved, social support, social networks, etc.). Moreover, care-recipient’s characteristics, such as gender, age, specific diseases, behavioral problems, types and quality of relationship, as well as co-residence are well known predictors of caregiver’s health [55, 56]. Therefore, further evidence on the causal effects of informal caregiving is required.

Second, the definition of an informal caregiver used in this study is broad and not specific enough to characterize by care-recipient’s disease such as dementia caregivers. For example, informal caregivers for people with dementia are shown to have higher level of stress, burden and depression than nondementia caregivers [57]. Relatedly, in studies of secondary data analysis of informal caregivers, defining informal caregiver varies by studies (e.g., self-reports on caregiving status without specific hourly criteria, providing care for at least 5 h per week, etc.) [22, 57, 58]. Hence, readers should keep in mind the definition of informal caregivers in this study when interpreting the results. Despite this, in supplementary analyses, we found that including those who provide at least one hour per week yields similar results to our findings about both main and gender effects (Table S6 in online supplementary material).

Third, this study was unable to consider several important health promoting behaviors such as sleep and dietary behaviors due mainly to data limitation. Future research may wish to investigate whether other health promoting behaviors may play a mediating role in the relationship between informal caregiving and health.

Fourth, this study did not directly investigate how engagement in informal caregiving affects caregiver’s time use and management, which this study implicitly considers as a reason for a reduction in physical exercise among women who became a caregiver during the survey period.

Lastly, because we didn’t use sampling weights in our estimation model, our results may not generate population-based estimates. However, applying longitudinal sampling weights is not ideal for our analytic strategy because our analysis is based on unbalanced panels, such that we must drop too many individuals (42% of the original sample) to apply sampling weights. Despite this limitation, we ran our core models using the KLoSA wave 7 longitudinal weights to examine how this affects our results (Table S7 in online supplementary material).

## Conclusions

Despite these limitations, to the best of our knowledge, this is the first study to directly test whether HPBs mediate the association between informal caregiving and subjective health. This study used a large-scale, nationally representative longitudinal dataset and used a rigorous methodological approach using longitudinal models. More specifically, to establish the temporal ordering of the variables and account for unobserved confounding factors, OLS-LDV models and FE models were used. This study also examined the importance of various health promoting behaviors in mediating the relationship between informal caregiving and subjective health. Importantly, the use of longitudinal data from a 12-year follow-up period enables us to identify a sufficient number of individuals who changed their caregiver status over time. This is crucial for this study’s analytic strategy.

Although providing informal care to family members can be a rewarding role, this labor is a serious threat to the subjective health of informal caregivers. One of the main findings of this study is that there are gender differences in the subjective health consequences of informal caregiving. This evidence suggests that programs that aim to improve the well-being of informal caregivers need to take a gender-conscious approach. More specifically, given the fact that insufficient physical activity is associated with chronic diseases, cognitive decline, and mortality [59, 60], the physical activity of informal caregivers should be monitored by medical staff of care-recipients and community services such as support groups for informal caregiver. Moreover, policy interventions to promote physical activity among women caregivers deserve increased priority.

## Supplementary Information

**Additional file 1.** 

## Data Availability

The datasets supporting the conclusions of this article is publicly available in https://survey.keis.or.kr/eng/klosa/klosa01.jsp.

## Abbreviations

ADL: Activities of daily living
FE: Fixed effects models
GEE: Generalized estimating equations
HPBs: Health promoting behaviors
HS: Health satisfaction
KLoSA: Korean Longitudinal Study of Aging
OLS-LDV: OLS regression models with lagged dependent variables
SRH: Self-rated health
