# Supplementary material for: Gender differences in the relationship between informal caregiving and subjective health: the mediating role of health promoting behaviors
Source: BMC Public Health. 2022 Feb 15;22:311. doi: 10.1186/s12889-022-12612-3 (PMC8845367; doi:10.1186/s12889-022-12612-3)
Supplement: Supplementary file 1 — Additional file 1. [file 12889_2022_12612_MOESM1_ESM.docx]

***Online Supplementary Material***

***Table S1. Regression of subjective health on informal caregiving, By gender and by estimation model with autoregressive correlation.***

| Gender | Full |  | Women |  | Men |  |
| --- | --- | --- | --- | --- | --- | --- |
| Estimation model | GEE | FE | GEE | FE | GEE | FE |
|  | DV | DV | DV | DV | DV | DV |
|  | at t+1 | at t | at t+1 | at t | at t+1 | at t |
|  | Model 1 | Model 2 | Model 3 | Model 4 | Model 5 | Model 6 |
| ***Panel A. DV = Health satisfaction*** |  |  |  |  |  |  |
| *Change in informal caregiving between t and t+1 (Ref: No to No)* |  |  |  |  |  |  |
| Started CG (No to Yes) | -1.389 |  | -2.664^*^ |  | 0.901 |  |
|  | [-3.119,0.341] |  | [-4.760,-0.569] |  | [-2.104,3.906] |  |
| Stopped CG (Yes to No) | -0.648 |  | 0.159 |  | -1.997 |  |
|  | [-2.334,1.038] |  | [-1.865,2.183] |  | [-4.992,0.997] |  |
| Continued CG (Yes to Yes) | 0.602 |  | -0.363 |  | 1.719 |  |
|  | [-2.524,3.727] |  | [-5.144,4.417] |  | [-2.121,5.558] |  |
| *Informal caregiving status at t* |  |  |  |  |  |  |
| Yes |  | -1.717 |  | -3.679^***^ |  | 2.099 |
|  |  | [-3.458,0.024] |  | [-5.849,-1.509] |  | [-0.656,4.853] |
| N | 7484 | 7484 | 4217 | 4217 | 3267 | 3267 |
| Observations | 39174 | 39174 | 22176 | 22176 | 16998 | 16998 |
| ***Panel B. DV = Self-rated health*** |  |  |  |  |  |  |
| *Change in informal caregiving between t and t+1 (Ref: No to No)* |  |  |  |  |  |  |
| Started CG (No to Yes) | -0.034 |  | -0.062^**^ |  | 0.012 |  |
|  | [-0.073,0.005] |  | [-0.108,-0.016] |  | [-0.056,0.080] |  |
| Stopped CG (Yes to No) | 0.020 |  | 0.024 |  | 0.020 |  |
|  | [-0.017,0.057] |  | [-0.021,0.069] |  | [-0.045,0.085] |  |
| Continued CG (Yes to Yes) | -0.083^**^ |  | -0.125^***^ |  | -0.033 |  |
|  | [-0.136,-0.029] |  | [-0.182,-0.067] |  | [-0.123,0.058] |  |
| *Informal caregiving status at t* |  |  |  |  |  |  |
| Yes |  | -0.025 |  | -0.055^*^ |  | 0.031 |
|  |  | [-0.062,0.011] |  | [-0.097,-0.013] |  | [-0.035,0.097] |
| N | 7490 | 7490 | 4220 | 4220 | 3270 | 3270 |
| Observations | 39211 | 39211 | 22196 | 22196 | 17015 | 17015 |
| Time-constant control variables | Yes | No | Yes | No | Yes | No |
| Time-varying control variables | Yes | Yes | Yes | Yes | Yes | Yes |
| Lagged dependent variable | Yes | No | Yes | No | Yes | No |

*Note*. Robust standard errors in GEE models and clustered standard errors in FE models were used. In the models, survey years were adjusted for. Time-constant control variables include gender, age, education, and number of children. Time-varying control variables include marital status, household size, household income, home owner, economic activity, region of residence, and number of chronic diseases. GEE = Generalized Estimating Equation, FE = Fixed Effects, DV = Dependent Variable, CG = Caregiver

* *p* < 0.05; ** *p* < 0.01; *** *p* < 0.001

***Table S2. Regression of mediating variables on informal caregiving, By gender with autoregressive correlation.***

|  | No  smoking at t+1 | No binge drinking at t+1 | Exercise at t+1 | Health check-up at t+1 |
| --- | --- | --- | --- | --- |
| Estimation model: GEE | Model 1 | Model 2 | Model 3 | Model 4 |
| ***Panel A. Women***  *Change in informal caregiving between t and t+1 (Ref: No to No)* |  |  |  |  |
| Started CG (No to Yes) | 0.010 | -0.007 | -0.498^***^ | -0.009 |
|  | [-0.008,0.028] | [-0.024,0.010] | [-0.736,-0.260] | [-0.067,0.049] |
| Stopped CG (Yes to No) | 0.002 | 0.007^*^ | 0.041 | -0.036 |
|  | [-0.003,0.007] | [0.000,0.014] | [-0.193,0.275] | [-0.084,0.013] |
| Continued CG (Yes to Yes) | -0.020 | -0.009 | -0.829^***^ | -0.068 |
|  | [-0.052,0.012] | [-0.039,0.021] | [-1.160,-0.497] | [-0.173,0.037] |
| N | 4221 | 4221 | 4221 | 4221 |
| Observations | 22202 | 22202 | 22202 | 22202 |
| ***Panel B. Men***  *Change in informal caregiving between t and t+1 (Ref: No to No)* |  |  |  |  |
| Started CG (No to Yes) | 0.064^*^ | 0.004 | 0.412 | -0.027 |
|  | [0.012,0.116] | [-0.045,0.053] | [-0.044,0.869] | [-0.099,0.045] |
| Stopped CG (Yes to No) | -0.020 | -0.013 | 0.042 | 0.043 |
|  | [-0.072,0.033] | [-0.062,0.035] | [-0.331,0.414] | [-0.013,0.099] |
| Continued CG (Yes to Yes) | 0.029 | 0.017 | 0.317 | 0.002 |
|  | [-0.036,0.094] | [-0.043,0.078] | [-0.342,0.976] | [-0.104,0.109] |
| N | 3270 | 3270 | 3270 | 3270 |
| Observations | 17015 | 17015 | 17015 | 17015 |
| Time-constant control variables | Yes | Yes | Yes | Yes |
| Time-varying control variables | Yes | Yes | Yes | Yes |
| Lagged dependent variable | Yes | Yes | Yes | Yes |

*Note*. Robust standard errors were used. In the models, survey years were adjusted for. Time-constant control variables include gender, age, education, and number of children. Time-varying control variables include marital status, household size, household income, home owner, economic activity, region of residence, and number of chronic diseases. GEE = Generalized Estimating Equation, CG = Caregiver.

* *p* < 0.05; ** *p* < 0.01; *** *p* < 0.001

***Table S3. Mediators linking informal caregiving to subjective health, Women only sample with autoregressive correlation.***

|  | DV at t+1 | DV at t+1 | DV at t+1 | DV at t+1 | DV at t+1 | DV at t+1 |
| --- | --- | --- | --- | --- | --- | --- |
| Estimation model: GEE | Model 1 | Model 2 | Model 3 | Model 4 | Model 5 | Model 6 |
| ***Panel A. DV = Health satisfaction*** |  |  |  |  |  |  |
| *Change in informal caregiving between t and t+1 (Ref: No to No)* |  |  |  |  |  |  |
| Started CG (No to Yes) | -2.664^*^ | -2.615^*^ | -2.656^*^ | -2.293^*^ | -2.621^*^ | -2.236^*^ |
|  | [-4.760,-0.569] | [-4.708,-0.521] | [-4.744,-0.569] | [-4.382,-0.204] | [-4.717,-0.525] | [-4.319,-0.154] |
| Stopped CG (Yes to No) | 0.159 | 0.145 | 0.159 | 0.335 | 0.218 | 0.362 |
|  | [-1.865,2.183] | [-1.883,2.173] | [-1.867,2.186] | [-1.675,2.346] | [-1.791,2.227] | [-1.641,2.365] |
| Continued CG (Yes to Yes) | -0.363 | -0.337 | -0.368 | 0.298 | -0.317 | 0.301 |
|  | [-5.144,4.417] | [-5.087,4.414] | [-5.147,4.411] | [-4.488,5.084] | [-5.018,4.384] | [-4.394,4.996] |
| *Change in health behaviors* |  |  |  |  |  |  |
| *between t and t+1* |  |  |  |  |  |  |
| No smoking |  | -0.915 |  |  |  | -1.355 |
|  |  | [-3.646,1.817] |  |  |  | [-4.090,1.379] |
| No binge drinking |  |  | 2.471^*^ |  |  | 2.043 |
|  |  |  | [0.226,4.715] |  |  | [-0.236,4.323] |
| Exercise |  |  |  | 0.512^***^ |  | 0.472^***^ |
|  |  |  |  | [0.398,0.626] |  | [0.358,0.587] |
| Health check-up |  |  |  |  | 2.683^***^ | 2.440^***^ |
|  |  |  |  |  | [2.040,3.325] | [1.800,3.079] |
| Mediation in “No to Yes” group |  |  |  |  |  |  |
| % total effect mediated | - | 2% | 0% | 14% | 2% | 16% |
| N | 4217 | 4217 | 4217 | 4217 | 4217 | 4217 |
| Observations | 22176 | 22176 | 22176 | 22176 | 22176 | 22176 |
| ***Panel B. DV = Self-rated health***  *Change in informal caregiving between t and t+1 (Ref: No to No)* |  |  |  |  |  |  |
| Started CG (No to Yes) | -0.062^**^ | -0.061^**^ | -0.062^**^ | -0.057^*^ | -0.062^**^ | -0.056^*^ |
|  | [-0.108,-0.016] | [-0.107,-0.016] | [-0.108,-0.016] | [-0.103,-0.011] | [-0.108,-0.016] | [-0.102,-0.011] |
| Stopped CG (Yes to No) | 0.024 | 0.024 | 0.024 | 0.024 | 0.024 | 0.024 |
|  | [-0.021,0.069] | [-0.021,0.068] | [-0.021,0.068] | [-0.020,0.069] | [-0.021,0.069] | [-0.021,0.068] |
| Continued CG (Yes to Yes) | -0.125^***^ | -0.124^***^ | -0.124^***^ | -0.116^***^ | -0.125^***^ | -0.116^***^ |
|  | [-0.182,-0.067] | [-0.181,-0.066] | [-0.182,-0.066] | [-0.174,-0.059] | [-0.183,-0.067] | [-0.173,-0.058] |
| *Change in health behaviors* |  |  |  |  |  |  |
| *between t and t+1* |  |  |  |  |  |  |
| No smoking |  | 0.015 |  |  |  | 0.013 |
|  |  | [-0.041,0.071] |  |  |  | [-0.043,0.069] |
| No binge drinking |  |  | 0.017 |  |  | 0.013 |
|  |  |  | [-0.035,0.070] |  |  | [-0.040,0.066] |
| Exercise |  |  |  | 0.009^***^ |  | 0.009^***^ |
|  |  |  |  | [0.006,0.011] |  | [0.006,0.011] |
| Health check-up |  |  |  |  | -0.002 | 0.005^***^ |
|  |  |  |  |  | [-0.015,0.011] | [0.003,0.008] |
| Mediation in “No to Yes” group |  |  |  |  |  |  |
| % total effect mediated | - | 2% | 0% | 8% | 0% | 10% |
| N | 4220 | 4220 | 4220 | 4220 | 4220 | 4220 |
| Observations | 22196 | 22196 | 22196 | 22196 | 22196 | 22196 |

*Note*. Robust standard errors were used. In GEE models, survey years were adjusted for. Time-constant control variables include gender, age, education, and number of children. Time-varying control variables include marital status, household size, household income, home owner, economic activity, region of residence, and number of chronic diseases. The table including level of mediating variables in Wave t is available in supplementary materials. GEE = Generalized Estimating Equation, DV = Dependent Variable, CG = Caregiver.

* *p* < 0.05; ** *p* < 0.01; *** *p* < 0.001

***Table S4. Logistic regression of self-rated health on informal caregiving, By gender.***

| Gender | Full |  |  | Women |  |  | Men |  |
| --- | --- | --- | --- | --- | --- | --- | --- | --- |
| Estimation model | GEE | FE |  | GEE | FE |  | GEE | FE |
|  | DV | DV |  | DV | DV |  | DV | DV |
|  | at t+1 | at t |  | at t+1 | at t |  | at t+1 | at t |
|  | Model 1 | Model 2 |  | Model 3 | Model 4 |  | Model 5 | Model 6 |
| ***Panel B. DV = Self-rated health*** |  |  |  |  |  |  |  |  |
| *Change in informal caregiving between t and t+1 (Ref: No to No)* |  |  |  |  |  |  |  |  |
| Started CG (No to Yes) | -0.364^**^ |  |  | -0.510^**^ |  |  | -0.171 |  |
|  | [-0.633,-0.094] |  |  | [-0.880,-0.141] |  |  | [-0.577,0.236] |  |
| Stopped CG (Yes to No) | 0.007 |  |  | 0.024 |  |  | 0.010 |  |
|  | [-0.209,0.222] |  |  | [-0.253,0.301] |  |  | [-0.337,0.357] |  |
| Continued CG (Yes to Yes) | -0.557^*^ |  |  | -1.158^**^ |  |  | -0.121 |  |
|  | [-0.999,-0.115] |  |  | [-1.999,-0.317] |  |  | [-0.689,0.447] |  |
| *Informal caregiving status at t* |  |  |  |  |  |  |  |  |
| Yes |  | -0.230 |  |  | -0.520^**^ |  |  | 0.150 |
|  |  | [-0.520,0.060] |  |  | [-0.915,-0.125] |  |  | [-0.286,0.586] |
| N | 9608 | 9608 |  | 5419 | 5419 |  | 4189 | 4189 |
| Observations | 42507 | 42507 |  | 24148 | 24148 |  | 18359 | 18359 |
| Time-constant control variables | Yes | No |  | Yes | No |  | Yes | No |
| Time-varying control variables | Yes | Yes |  | Yes | Yes |  | Yes | Yes |
| Lagged dependent variable | Yes | No |  | Yes | No |  | Yes | No |

*Note*. Robust standard errors in GEE models and clustered standard errors in FE models were used. In GEE models, survey years were adjusted for. Time-constant control variables include gender, age, education, and number of children. Time-varying control variables include marital status, household size, household income, home owner, economic activity, region of residence, and number of chronic diseases. The table including level of mediating variables in Wave t is available in supplementary materials. GEE = Generalized Estimating Equation, FE = Fixed Effects, DV = Dependent Variable, CG = Caregiver.

* *p* < 0.05; ** *p* < 0.01; *** *p* < 0.001

***Table S5. Mediators linking informal caregiving to subjective health, Women only sample.***

|  | DV at t+1 | DV at t+1 | DV at t+1 | DV at t+1 | DV at t+1 | DV at t+1 |
| --- | --- | --- | --- | --- | --- | --- |
| Estimation model: GEE | Model 1 | Model 2 | Model 3 | Model 4 | Model 5 | Model 6 |
| ***Panel A. DV = Health satisfaction*** |  |  |  |  |  |  |
| *Change in informal caregiving between t and t+1 (Ref: No to No)* |  |  |  |  |  |  |
| Started CG (No to Yes) | -3.236^**^ | -3.193^**^ | -3.238^**^ | -2.845^**^ | -3.160^**^ | -2.763^**^ |
|  | [-5.318,-1.154] | [-5.274,-1.112] | [-5.316,-1.160] | [-4.925,-0.764] | [-5.242,-1.078] | [-4.839,-0.688] |
| Stopped CG (Yes to No) | -0.590 | -0.596 | -0.593 | -0.398 | -0.501 | -0.343 |
|  | [-2.431,1.252] | [-2.440,1.249] | [-2.437,1.250] | [-2.234,1.438] | [-2.338,1.335] | [-2.178,1.491] |
| Continued CG (Yes to Yes) | -1.948 | -1.953 | -1.943 | -1.437 | -1.894 | -1.422 |
|  | [-6.713,2.817] | [-6.691,2.785] | [-6.705,2.819] | [-6.216,3.341] | [-6.629,2.841] | [-6.150,3.307] |
| *Change in health behaviors* |  |  |  |  |  |  |
| *between t and t+1* |  |  |  |  |  |  |
| No smoking |  | -0.360 |  |  |  | -0.852 |
|  |  | [-2.940,2.220] |  |  |  | [-3.437,1.732] |
| No binge drinking |  |  | 1.980 |  |  | 1.641 |
|  |  |  | [-0.245,4.205] |  |  | [-0.599,3.882] |
| Exercise |  |  |  | 0.552^***^ |  | 0.516^***^ |
|  |  |  |  | [0.445,0.659] |  | [0.409,0.623] |
| Health check-up |  |  |  |  | 2.650^***^ | 2.404^***^ |
|  |  |  |  |  | [2.037,3.263] | [1.794,3.013] |
| Mediation in “No to Yes” group |  |  |  |  |  |  |
| % total effect mediated | - | 1% | 0% | 12% | 2% | 15% |
| N | 5,418 | 5,418 | 5,418 | 5,418 | 5,418 | 5,418 |
| Observations | 24,135 | 24,135 | 24,135 | 24,135 | 24,135 | 24,135 |
| ***Panel B. DV = Self-rated health***  *Change in informal caregiving between t and t+1 (Ref: No to No)* |  |  |  |  |  |  |
| Started CG (No to Yes) | -0.063^**^ | -0.063^**^ | -0.063^**^ | -0.058^*^ | -0.064^**^ | -0.058^*^ |
|  | [-0.109,-0.018] | [-0.108,-0.018] | [-0.108,-0.018] | [-0.103,-0.013] | [-0.109,-0.018] | [-0.103,-0.013] |
| Stopped CG (Yes to No) | 0.003 | 0.003 | 0.002 | 0.004 | 0.002 | 0.003 |
|  | [-0.037,0.042] | [-0.037,0.042] | [-0.038,0.042] | [-0.036,0.043] | [-0.037,0.042] | [-0.037,0.042] |
| Continued CG (Yes to Yes) | -0.120^***^ | -0.119^***^ | -0.119^***^ | -0.113^***^ | -0.120^***^ | -0.112^***^ |
|  | [-0.180,-0.060] | [-0.179,-0.059] | [-0.180,-0.059] | [-0.173,-0.053] | [-0.180,-0.060] | [-0.172,-0.052] |
| *Change in health behaviors* |  |  |  |  |  |  |
| *between t and t+1* |  |  |  |  |  |  |
| No smoking |  | 0.025 |  |  |  | 0.022 |
|  |  | [-0.026,0.077] |  |  |  | [-0.029,0.073] |
| No binge drinking |  |  | 0.025 |  |  | 0.021 |
|  |  |  | [-0.027,0.076] |  |  | [-0.030,0.072] |
| Exercise |  |  |  | 0.009^***^ |  | 0.009^***^ |
|  |  |  |  | [0.007,0.012] |  | [0.007,0.012] |
| Health check-up |  |  |  |  | -0.006 | -0.010 |
|  |  |  |  |  | [-0.019,0.006] | [-0.022,0.002] |
| Mediation in “No to Yes” group |  |  |  |  |  |  |
| % total effect mediated | - | 0% | 0% | 8% | -2% | 8% |
| N | 5,419 | 5,419 | 5,419 | 5,419 | 5,419 | 5,419 |
| Observations | 24,148 | 24,148 | 24,148 | 24,148 | 24,148 | 24,148 |

*Note*. Robust standard errors were used. In GEE models, survey years were adjusted for. Time-constant control variables include gender, age, education, and number of children. Time-varying control variables include marital status, household size, household income, home owner, economic activity, region of residence, and number of chronic diseases. The table including level of mediating variables in Wave t is available in supplementary materials. GEE = Generalized Estimating Equation, DV = Dependent Variable, CG = Caregiver.

* *p* < 0.05; ** *p* < 0.01; *** *p* < 0.001

***Table S6. Regression of subjective health on informal caregiving, By gender and all informal caregivers (providing informal care more than 1 hour per week)***

| Gender | Full |  |  | Women |  |  | Men |  |
| --- | --- | --- | --- | --- | --- | --- | --- | --- |
| Estimation model | GEE | FE |  | GEE | FE |  | GEE | FE |
|  | DV | DV |  | DV | DV |  | DV | DV |
|  | at t+1 | at t |  | at t+1 | at t |  | at t+1 | at t |
|  | Model 1 | Model 2 |  | Model 3 | Model 4 |  | Model 5 | Model 6 |
| ***Panel A. DV = Health satisfaction*** |  |  |  |  |  |  |  |  |
| *Change in informal caregiving between t and t+1 (Ref: No to No)* |  |  |  |  |  |  |  |  |
| Started CG (No to Yes) | -2.757^***^ |  |  | -3.199^***^ |  |  | -1.976 |  |
|  | [-4.248,-1.267] |  |  | [-4.955,-1.443] |  |  | [-4.612,0.660] |  |
| Stopped CG (Yes to No) | -0.778 |  |  | -0.482 |  |  | -1.130 |  |
|  | [-2.183,0.627] |  |  | [-2.180,1.215] |  |  | [-3.587,1.327] |  |
| Continued CG (Yes to Yes) | -0.987 |  |  | -3.045 |  |  | 1.539 |  |
|  | [-3.705,1.731] |  |  | [-6.911,0.821] |  |  | [-1.980,5.058] |  |
| *Informal caregiving status at t* |  |  |  |  |  |  |  |  |
| Yes |  | -2.173^**^ |  |  | -3.282^***^ |  |  | -0.125 |
|  |  | [-3.665,-0.680] |  |  | [-5.130,-1.434] |  |  | [-2.567,2.317] |
| N | 9606 | 9606 |  | 5418 | 5418 |  | 4188 | 4188 |
| Observations | 42482 | 42482 |  | 24135 | 24135 |  | 18347 | 18347 |
| ***Panel B. DV = Self-rated health*** |  |  |  |  |  |  |  |  |
| *Change in informal caregiving between t and t+1 (Ref: No to No)* |  |  |  |  |  |  |  |  |
| Started CG (No to Yes) | -0.111^**^ |  |  | -0.127^**^ |  |  | -0.084 |  |
|  | [-0.181,-0.042] |  |  | [-0.215,-0.040] |  |  | [-0.198,0.030] |  |
| Stopped CG (Yes to No) | 0.014 |  |  | -0.002 |  |  | 0.050 |  |
|  | [-0.041,0.068] |  |  | [-0.071,0.066] |  |  | [-0.039,0.139] |  |
| Continued CG (Yes to Yes) | -0.085 |  |  | -0.105 |  |  | -0.045 |  |
|  | [-0.184,0.015] |  |  | [-0.226,0.016] |  |  | [-0.206,0.115] |  |
| *Informal caregiving status at t* |  |  |  |  |  |  |  |  |
| Yes |  | -0.076^*^ |  |  | -0.111^**^ |  |  | -0.013 |
|  |  | [-0.135,-0.018] |  |  | [-0.180,-0.042] |  |  | [-0.115,0.090] |
| N | 9608 | 9608 |  | 5419 | 5419 |  | 4189 | 4189 |
| Observations | 42507 | 42507 |  | 24148 | 24148 |  | 18359 | 18359 |
| Time-constant control variables | Yes | No |  | Yes | No |  | Yes | No |
| Time-varying control variables | Yes | Yes |  | Yes | Yes |  | Yes | Yes |
| Lagged dependent variable | Yes | No |  | Yes | No |  | Yes | No |

*Note*. Robust standard errors in GEE models and clustered standard errors in FE models were used. In GEE models, survey years were adjusted for. Time-constant control variables include gender, age, education, and number of children. Time-varying control variables include marital status, household size, household income, home owner, economic activity, region of residence, and number of chronic diseases. The table including level of mediating variables in Wave t is available in supplementary materials. GEE = Generalized Estimating Equation, FE = Fixed Effects, DV = Dependent Variable, CG = Caregiver.

* *p* < 0.05; ** *p* < 0.01; *** *p* < 0.001

***Table S7. Regression of subjective health on informal caregiving, By gender and by estimation model with weighted sample.***

| Gender | Full |  | Women |  | Men |  |
| --- | --- | --- | --- | --- | --- | --- |
| Estimation model | GEE | FE | GEE | FE | GEE | FE |
|  | DV | DV | DV | DV | DV | DV |
|  | at t+1 | at t | at t+1 | at t | at t+1 | at t |
|  | Model 1 | Model 2 | Model 3 | Model 4 | Model 5 | Model 6 |
| ***Panel A. DV = Health satisfaction*** |  |  |  |  |  |  |
| *Change in informal caregiving between t and t+1 (Ref: No to No)* |  |  |  |  |  |  |
| Started CG (No to Yes) | -1.509 |  | -3.605^*^ |  | 1.978 |  |
|  | [-3.957,0.939] |  | [-6.533,-0.678] |  | [-2.177,6.134] |  |
| Stopped CG (Yes to No) | -1.179 |  | -0.878 |  | -1.493 |  |
|  | [-3.091,0.733] |  | [-3.162,1.405] |  | [-4.934,1.947] |  |
| Continued CG (Yes to Yes) | -5.770^*^ |  | -8.190^*^ |  | -2.142 |  |
|  | [-10.229,-1.311] |  | [-14.739,-1.641] |  | [-7.160,2.876] |  |
| *Informal caregiving status at t* |  |  |  |  |  |  |
| Yes |  | -2.942^*^ |  | -5.126^***^ |  | 0.968 |
|  |  | [-5.183,-0.702] |  | [-7.784,-2.469] |  | [-2.844,4.781] |
| N | 5578 | 5578 | 3241 | 3241 | 2337 | 2337 |
| Observations | 32677 | 32677 | 18861 | 18861 | 13816 | 13816 |
| ***Panel B. DV = Self-rated health*** |  |  |  |  |  |  |
| *Change in informal caregiving between t and t+1 (Ref: No to No)* |  |  |  |  |  |  |
| Started CG (No to Yes) | -0.044 |  | -0.069^*^ |  | -0.000 |  |
|  | [-0.097,0.010] |  | [-0.128,-0.010] |  | [-0.100,0.099] |  |
| Stopped CG (Yes to No) | 0.026 |  | 0.023 |  | 0.033 |  |
|  | [-0.021,0.072] |  | [-0.033,0.079] |  | [-0.049,0.116] |  |
| Continued CG (Yes to Yes) | -0.135^**^ |  | -0.193^***^ |  | -0.045 |  |
|  | [-0.217,-0.053] |  | [-0.261,-0.125] |  | [-0.208,0.117] |  |
| *Informal caregiving status at t* |  |  |  |  |  |  |
| Yes |  | -0.031 |  | -0.079^**^ |  | 0.053 |
|  |  | [-0.077,0.015] |  | [-0.130,-0.029] |  | [-0.031,0.136] |
| N | 5578 | 5578 | 3241 | 3241 | 2337 | 2337 |
| Observations | 32693 | 32693 | 18871 | 18871 | 13822 | 13822 |
| Time-constant control variables | Yes | No | Yes | No | Yes | No |
| Time-varying control variables | Yes | Yes | Yes | Yes | Yes | Yes |
| Lagged dependent variable | Yes | No | Yes | No | Yes | No |

*Note*. The KLoSA wave 7 longitudinal weights were used. Robust standard errors were used. In the models, survey years were adjusted for. Time-constant control variables include gender, age, education, and number of children. Time-varying control variables include marital status, household size, household income, home owner, economic activity, region of residence, and number of chronic diseases. GEE = Generalized Estimating Equation, FE = Fixed Effects, DV = Dependent Variable, CG = Caregiver

* *p* < 0.05; ** *p* < 0.01; *** *p* < 0.001
